# Impact of *CYP19A1* and *ESR1* variants on early-onset side effects during combined endocrine therapy in the TEXT trial

**DOI:** 10.1186/s13058-016-0771-8

**Published:** 2016-11-08

**Authors:** Harriet Johansson, Kathryn P. Gray, Olivia Pagani, Meredith M. Regan, Giuseppe Viale, Valentina Aristarco, Debora Macis, Antonella Puccio, Susanne Roux, Rudolf Maibach, Marco Colleoni, Manuela Rabaglio, Karen N. Price, Alan S. Coates, Richard D. Gelber, Aron Goldhirsch, Roswitha Kammler, Bernardo Bonanni, Barbara A. Walley, the TEXT principal investigators

**Affiliations:** 1 Division of Cancer Prevention and Genetics, European Institute of Oncology, Via Ripamonti 435, Milan, 20141 Italy; 2 International Breast Cancer Study Group (IBCSG) Statistical Center, Department of Biostatistics and Computational Biology, Dana-Farber Cancer Institute, Harvard T. H. Chan School of Public Health, 450 Brookline Avenue, Boston, MA 02215 USA; 3 Institute of Oncology of Southern Switzerland (IOSI), Bellinzona, Switzerland; 4 International Breast Cancer Study Group, Bern, Switzerland; 5 Swiss Group for Clinical Cancer Research SAKK, Lugano Viganello, Switzerland; 6 IBCSG Statistical Center, Department of Biostatistics and Computational Biology, Dana-Farber Cancer Institute, Harvard Medical School, 450 Brookline Avenue, Boston, MA 02215 USA; 7 Department of Pathology and Laboratory Medicine, IBCSG Central Pathology Laboratory, European Institute of Oncology, and University of Milan, Via Ripamonti 435, Milan, 20141 Italy; 8 International Breast Cancer Study Group (IBCSG) Coordinating Center, Effingerstrasse 40, Bern, CH-3008 Switzerland; 9 Division of Medical Senology, European Institute of Oncology, Via Ripamonti 435, Milan, 20141 Italy; 10 IBCSG Statistical Center, Frontier Science and Technology Research Foundation, Boston, MA USA; 11 IBCSG Statistical Center, Frontier Science and Technology Research Foundation, Boston, MA USA; 12 International Breast Cancer Study Group and University of Sydney School of Public Health, Sydney, Australia; 13 IBCSG Statistical Center, Department of Biostatistics and Computational Biology, Dana-Farber Cancer Institute, Harvard T.H. Chan School of Public Health, Harvard Medical School, Frontier Science and Technology Research Foundation, 450 Brookline Avenue, Boston, MA 02215 USA; 14 Program for Breast Health, European Institute of Oncology, Via Ripamonti 435, Milan, 20141 Italy; 15 Translational Research Coordination and Central Pathology Office, IBCSG Coordinating Center, Effingerstrasse 40, Bern, CH-3008 Switzerland; 16 Breast Unit of Southern Switzerland, Bellinzona, Switzerland; 17 National Cancer Institute of Canada, Kingston, ON Canada; 18 Dana-Farber Cancer Institute, Department of Biostatistics and Computatonal Biology, 450 Brookline Ave, Boston, MA 02215 USA

**Keywords:** Side effects, Aromatase inhibitors, Tamoxifen, Ovarian suppression, Breast cancer, *CYP19A1*, *ESR1*

## Abstract

**Background:**

Single nucleotide polymorphisms (SNPs) in the estrogen receptor 1 (*ESR1*) and cytochrome P450 19A1 (*CYP19A1*) genes have been associated with breast cancer risk, endocrine therapy response and side effects, mainly in postmenopausal women with early breast cancer. This analysis aimed to assess the association of selected germline *CYP19A1* and *ESR1* SNPs with early-onset hot flashes, sweating and musculoskeletal symptoms in premenopausal patients enrolled in the Tamoxifen and Exemestane Trial (TEXT).

**Methods:**

Blood was collected from consenting premenopausal women with hormone-responsive early breast cancer, randomly assigned to 5-years of tamoxifen plus ovarian suppression (OFS) or exemestane plus OFS. DNA was extracted with QIAamp kits and genotyped for two *CYP19A1* (rs4646 and rs10046) and three *ESR1* (rs2077647, rs2234693 and rs9340799) SNPs by a real-time pyrosequencing technique. Adverse events (AEs) were recorded at baseline and 3-monthly during the first year. Associations of the genotype variants with grade ≥2 early-onset targeted AEs of hot flashes/sweating or musculoskeletal events were assessed using logistic regression models.

**Results:**

There were 2660 premenopausal patients with breast cancer in the intention-to-treat population of TEXT, and 1967 (74 %) are included in this translational study. The *CYP19A1* rs10046 variant T/T, represented in 23 % of women, was associated with a reduced incidence of grade ≥2 hot flashes/sweating (univariate odds ratio (OR) = 0.78; 95 % CI 0.63–0.97; *P* = 0.03), more strongly in patients assigned exemestane + OFS (TT vs CT/CC: OR = 0.65, 95 % CI = 0.48–0.89) than assigned tamoxifen + OFS (OR = 0.94, 95 % CI = 0.69–1.27, interaction *P* = 0.03). No association with any of the *CYP19A1/ESR1* genotypes and musculoskeletal AEs was found.

**Conclusion:**

The *CYP19A1* rs10046 variant T/T favors lower incidence of hot flashes/sweating under exemestane + OFS treatment, suggesting endocrine-mediated effects. Based on findings from others, this SNP may potentially enhance treatment adherence and treatment efficacy. We plan to evaluate the clinical impact of this polymorphism during time, pending sufficient median follow up.

**Trial registration:**

ClinicalTrials.gov NCT00066703, registered August 6, 2003.

## Background

Adjuvant endocrine therapy significantly prolongs disease-free and overall survival in women with hormone-receptor-positive early breast cancer, but it is associated with several side effects, which may lead to early treatment cessation [1–3]. In the combined analysis of the Tamoxifen and Exemestane Trial (TEXT) and Suppression of Ovarian Function Trial (SOFT) [4], comparing adjuvant exemestane plus ovarian function suppression (OFS) with tamoxifen plus OFS in premenopausal patients with breast cancer, early cessation of OFS and the assigned oral endocrine treatment occurred in 16 % of patients receiving exemestane + OFS and 11 % of those receiving tamoxifen + OFS. Nonetheless, exemestane + OFS significantly improved disease outcome compared to tamoxifen + OFS after 5.7 years median follow up.

The acute onset of menopause induced by gonadotropin-releasing-hormone analogues (GnRHa) is associated with more frequent and severe side effects compared to natural menopause, significantly impacting the quality of life of young patients with breast cancer [5]. The most common side effects associated with early menopause include vasomotor symptoms (hot flashes and sweating), decreased libido, insomnia, and dyspareunia secondary to vaginal dryness. The frequency and severity of hot flashes may depend on the abrupt fall in circulating estrogen levels as observed in several studies among women undergoing a natural menopausal transition [6–8], although other factors also play a role [9, 10]. While chemotherapy, OFS, and aromatase inhibitors (AIs) directly lower circulating estrogen levels, tamoxifen, a selective estrogen receptor modulator, has both agonistic and antagonistic effects on estrogen signaling [11]. In addition to menopausal symptoms, AIs are frequently associated with joint and muscle pain [12], decreased bone density [13] and risk of fracture [3], which appears to increase with better compliance with AIs [14].

Common genetic polymorphisms of the genes involved in estrogen production and estrogen target genes have been linked to breast cancer risk, prognosis, treatment response and side effects. One of these genes, the *CYP19A1,* encodes for the enzyme aromatase that promotes the bioconversion of androgens to estrogens. Genetic variations at the *CYP19A1* locus may result in increased or decreased aromatase activity and influence concentrations of circulating estrogens [15–17]. For example, the rs10046 and rs4646 variants, located in a 3’ untranslated region, were associated with higher estradiol and estrone levels due to increased aromatase activity. Alternatively, these variants could be linked with other gene variants such as the rs749292, which is associated with even higher estrogen levels [17]. A recent review and meta-analysis analyzed the influence of common *CYP19A1* polymorphisms on postmenopausal patients with breast cancer treated with AIs [18], indicating a certain heterogeneity between studies.

The estrogen receptor α (*ESR1)* gene was recently recognized as a low-penetrance breast cancer susceptibility gene. Numerous studies suggest an association between *ESR1* gene polymorphisms and breast cancer risk [19]. However, results have been controversial due to heterogeneous data sources, differences in study designs, ethnic background, disease status, and sample size. *ESR1* is an important mediator of endocrine pathways involved in breast cancer risk and outcomes, including endocrine treatment response and side effects. Genetic polymorphisms altering the expression of *ESR1* have been suggested to affect breast cancer susceptibility [20]. In particular, the restriction enzymes XbaI (rs9340799) and PvuII (rs2234693) have been extensively evaluated. Both are located in the first intron of the *ESR1* gene. The association between variant allele T of *ESR1* PvuII (C > T) and breast cancer appears to be linked to a higher transcriptional activity of the variant gene [21] and correlated with circulating estrogen levels [22].

A recent meta-analysis [23] found that menopausal status modifies breast cancer risk associated with *ESR1* PvuII (C > T), with premenopausal variant carriers being at higher risk, possibly related to differences in circulating estrogen levels [22]. However, an updated meta-analysis restricted the effect to the Asian population [24]. Another meta-analysis of almost 19,000 individuals in eight European centers reported that *ESR1* XbaI (A > G) protects against overall fracture risk [25], suggesting an involvement of these polymorphisms in bone metabolism. These *ESR1* polymorphisms have also been described to be involved in ovarian hyperstimulation response in assisted reproduction studies [26], further highlighting their role in endocrine-related mechanisms.

Within the phase III TEXT trial in which 2672 premenopausal women were randomized to adjuvant therapy with exemestane + OFS or tamoxifen + OFS, with or without adjuvant chemotherapy, we prospectively designed a translational research project for blood collection to investigate the effect of selected single nucleotide polymorphisms (SNPs) on treatment efficacy and toxicity. The purpose of the current analysis was to investigate the association of common genetic variants of *CYP19A1* (rs10046, rs4646) and *ESR1* (rs2077647, rs2234693 and rs9340799) with early-onset vasomotor and musculoskeletal symptoms.

## Methods

### Patients

TEXT is an International Breast Cancer Study Group (IBCSG)-coordinated, randomized, phase III trial that enrolled premenopausal women with histologically proven estrogen receptor (ER) and/or progesterone receptor (PgR)-positive early breast cancer. From November 2003 through April 2011, patients were enrolled within 12 weeks from surgery, prior to the initiation of any systemic adjuvant therapy, and randomized to 5 years of exemestane + OFS or tamoxifen + OFS. OFS was achieved by monthly injection of the GnRHa triptorelin; bilateral oophorectomy or ovarian irradiation was allowed after at least 6 months of triptorelin. Chemotherapy was optional and, if administered, triptorelin and chemotherapy were started concomitantly; oral endocrine treatment was started after the completion of chemotherapy, or if chemotherapy was not administered, it was started 6 to 8 weeks after the initiation of triptorelin, to allow for the suppression of ovarian estrogen production.

### Trial procedures

Targeted adverse events (AEs) were systematically collected, using the Common Terminology Criteria for Adverse Events (CTCAE) Version 3.0, at baseline and every 3 months during the first year of treatment: hot flashes was graded 1–3 (1, mild; 2, moderate; 3, interfering with activities of daily living (ADL)); sweating was graded 1–2 (1, mild and occasional; 2, frequent or drenching); and musculoskeletal symptoms, i.e., myalgia, arthralgia (joint pain), stiffness, were graded 1–4 (1, mild pain, not interfering with function; 2, moderate pain, pain or analgesics interfering with function but not interfering with ADL; 3, severe pain, pain or analgesics severely interfering with ADL; 4, disabling). Depending on institutional procedures, patients may have been systematically asked about targeted AEs during the clinical visit, or targeted AEs may have been recorded in the CRFs based on review of the medical reports.

Protocol amendment 2, dated July 2008, increased the sample size and added the collection of a single whole blood sample for DNA isolation for translational research objectives, i.e., to investigate treatment tolerability and disease outcome according to genetic polymorphisms. Samples and consent were prospectively collected for approximately 600 patients enrolled after the amendment, and approximately 2000 patients enrolled prior to the amendment were asked to re-consent and have samples collected at the next scheduled protocol visit. The translational protocol targeted collection was of 2000 total samples.

### Blood collection, DNA extraction and genotyping assays

Venous blood was collected into EDTA-treated tubes provided by IBCSG and either processed and stored locally at −80 °C or shipped immediately to the CALGB Pathology Coordinating Office (USA and Canada), for DNA extraction and temporary storage, until shipping to the IBCSG central biomarker laboratory at the European Institute of Oncology for biobanking, DNA extraction (all countries except USA and Canada) and genotyping. Genomic DNA was extracted with QIAamp DNA Blood Kits (Qiagen, Valencia, CA, USA), according to the manufacturer’s instructions and extraction was performed by the automated platform “QIAcube” (Qiagen, Valencia, CA, USA).

The germline DNA samples were genotyped for SNPs in *CYP19A1* (rs4646 and rs10046) and *ESR1* (rs207764, rs2234693 and rs9340799). All samples were analyzed using a real-time sequencing method called pyrosequencing (Diatech Pharmacogenetics S.r.l., Jesi, Italy). The DNA was amplified by polymerase chain reaction (PCR) with biotinylated primers on the Real-Time PCR Cycler “Rotor-Gene TM 6000” (Corbett Research, Sydney, Australia), whereas single-stranded DNA templates were prepared using the PyroMark Vacuum Prep Workstation (Biotage, Uppsala, Sweden). The pyrosequencing analysis was performed on the PyroMarkTM Q96 ID instrument (Biotage). Control samples, representing a complete set of genotypes (wt/wt; wt/v; v/v) for all SNPs, were processed in each run. No patient sample failed genotyping.

### Statistical analysis

**Fig. 1:**
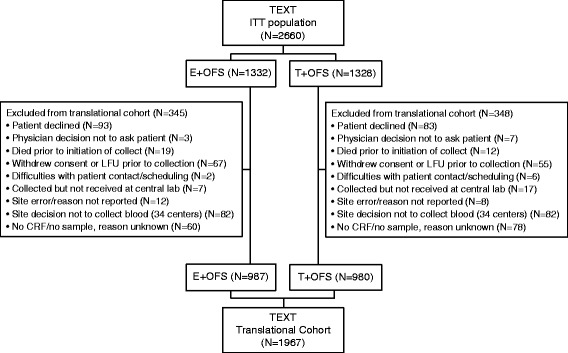
Derivation of the Tamoxifen and Exemestane Trial (*TEXT*) translational cohort from the intention-to-treat (*ITT*) population. The translational cohort includes patients whose blood was available for DNA analysis. *GNRHa* triptorelin was required for the first 6 months, any time after which the patient could choose to undergo bilateral oophorectomy or bilateral ovarian radiotherapy. *E* exemestane, *T* tamoxifen, *OFS* ovarian function suppression, *LFU* lost to follow up, *CRF* case report form

The analysis included 1967 patients from the TEXT intention-to-treat population (ITT) who gave whole blood for genetic profiling (Fig. 1). The endpoint of early-onset hot flushes/sweating was defined as presence or absence of grade 2 or grade 3 hot flashes or grade 2 sweating reported at the 3 month or 6 month visits after randomization. Early-onset musculoskeletal symptoms were defined as presence or absence of grade 2–4 musculoskeletal symptoms reported at the visits at 3, 6, 9 or 12 months after randomization.

Logistic regression modeling assessed the association of the selected genotypes with presence of early-onset AEs. The model also adjusted for patient characteristics at randomization: age (<45 versus ≥45 years); menstruation status (normal versus irregular versus persistent amenorrhea); body mass index (BMI) (normal (<25), overweight (25–29.9) versus obese (≥30) kg/m^2^)); adjuvant chemotherapy use (yes versus no); treatment assignment (exemestane + OFS versus tamoxifen + OFS); presence of hot flashes/sweating of any grade at baseline; and presence of musculoskeletal symptoms of any grade at baseline. Because concomitant medications may affect the reported AE severity, the impact of relevant concomitant medication use (yes versus no) prior to or continuing at baseline, or introduced during the relevant endpoint time period, was investigated in a sensitivity analysis. Concomitant medications, prescribed for any reason, that might affect the severity of hot flashes/sweating included venlafaxine, SSRIs, clonidine, gabapentin, pregabalin and herbals [27]; medications for musculoskeletal symptoms such as non-steroidal anti-inflammatory drugs (NSAIDs), glucosamine, corticosteroids, gabapentin, and pregabalin. The analyses also assessed whether the association varied by treatment assignment by including genotype variants-by-treatment interaction in the logistic regression models.

We first assessed SNP variant effects in an additive genotype model that compared 0 versus 1 versus 2 minor or variant alleles using a one-degree-of-freedom trend test. The minor or variant homozygote effect was assessed in a recessive model that compared the minor or variant homozygote versus the combined heterozygote and wild-type homozygote (reference group) using the chi-squared test. Hardy-Weinberg equilibrium (HWE) for genotype frequencies was tested using the Monte Carlo simulation method [28] to calculate the *P* value in order to avoid the reliance on the underlying chi-square approximation.

The study is presented in accordance with the Reporting Recommendations for Tumor Marker Prognostic Studies (REMARK) criteria [29]. All statistical tests were two-sided, without adjustment for multiple comparisons, and a *P* value <0.05 in the overall cohort or interaction *P* value ≤0.10 was considered as statistically significant. For a given sample size, assuming 10 % or 20 % homozygous variant and 43 % and 26 % AE rates, the detectable differences in AE rates for homozygous versus combined heterozygous and wild-type would be in the range of 11 % to 7.2 % (Fisher’s exact test, two-sided α = 0.05, power ≥80 %).

## Results

### Study population

Blood for germline DNA extraction from 1967 consenting women was collected and assessed, representing 74 % of the entire TEXT ITT population of 2660 (Fig. 1). Patients in the analytical cohort were representative of the TEXT trial (Table 1), with the exceptions of race (one country did not participate and some centers with a majority of hispanic ethnicity had low participation rates) and early discontinuation of protocol treatment (retrospective nature of sample collection). Most patients were Caucasian (92 %), median age was 44 years and median body mass index was 24 kg/m^2^. Adjuvant chemotherapy was given to 58 % of patients.

**Table 1:** Characteristics of TEXT intention-to-treat population, overall and according to availability of blood for DNA analysis

	Blood for DNA analysis		TEXT population (*n* = 2660)
	No (*n* = 693)	Yes (*n* = 1967)	
Characteristics at randomization			
White/Caucasian,%	73	92	87
Age (years), median (IQR)	43 (39, 46)	44 (40, 47)	43 (40, 46)
Normal menstruation, %	87	88	87
BMI (kg/m^2^), median (IQR)	24 (22, 29)	24 (21, 28)	24 (21, 28)
Presence of any grade (1–3) hot flashes, %	5	8	7
Presence of any grade (1–2) sweating, %	4	7	6
Presence of any grade (1–4) musculoskeletal symptoms, %	13	15	15
Concomitant adjuvant therapy			
Adjuvant chemotherapy, %	64	58	59
HER2-directed therapy, %	3	7	6
Protocol adjuvant therapy			
Treatment assignment			
Exemestane + OFS, %	50	50	50
Tamoxifen + OFS, %	50	50	50
Oral endocrine therapy (exemestane or tamoxifen) treatment <12 months, %	19	6	10
OFS <12 months, %	16	4	7
Analysis endpoints^a^			
Early-onset grade ≥2 hot flashes/sweating, %	41	43	43
Early-onset grade ≥2 musculoskeletal symptoms, %	28	26	27

^a^Adverse events according to common terminology criteria for adverse events (CTCAE) v3.0 of hot flashes and/or sweating reported at 3 or 6 months after randomization; musculoskeletal symptoms, i.e., myalgia, arthralgia (joint pain), or stiffness, reported at 3, 6, 9 or 12 months after randomization. *TEXT* Tamoxifen and Exemestane Trial, *BMI* body mass index, *IQR* interquartile range, *HER2* human epidermal growth factor receptor 2, *OFS* ovarian function suppression

At baseline, any grade (≥1) of hot flashes and sweating were reported in 8 % and 7 % of patients, respectively, while any grade (≥1) of musculoskeletal symptoms were reported in 15 % of patients (Table 1).

The reference SNP numbers, minor allele frequencies and genotype frequencies for each analyzed SNP are listed in Table 2. No deviations from Hardy-Weinberg equilibrium were observed. Occurrence and grade of hot flashes, sweating and musculoskeletal side effects during the first year of protocol therapy, overall and by treatment assignment, are depicted in Table 3.

**Table 2:** Minor allele frequency and genotype of the five genotyped SNPs in *CYP19A1* and *ESR1*

				Genotype, *n* (%)			
Gene	SNP	Number assessed	Minor allele frequency	Wild-type	Heterozygous	Homozygous	HWE *P* value
*CYP19A1*	rs4646 (G > T)	1967	0.29	989 (50)	822 (42)	156 (8)	0.44
*CYP19A1*	rs10046 (C > T)	1967	0.48	532 (27)	989 (50)	446 (23)	0.75
*ESR1*	rs2077647 (A > G)	1967	0.47	550 (28)	999 (51)	418 (21)	0.39
*ESR1*	rs2234693 (Pvull) (T > C)	1967	0.45	594 (30)	993 (50)	380 (19)	0.36
*ESR1*	rs9340799 (Xbal) (A > G)	1967	0.36	806 (41)	923 (47)	238 (12)	0.30

*CYP19A1* Cytochrome P450 19A1, *ESR1* Estrogen receptor 1, *HWE* Hardy-Weinberg equilibrium, *SNP* single nucleotide polymorphism

**Table 3:** Analysis endpoints and side effects during first year of protocol therapy according to treatment assignment

			Treatment	
		Overall	Exemestane + OFS	Tamoxifen + OFS
		(*n* = 1967^a^)	(*n* = 987)	(*n* = 980)
Analysis endpoint				
Early-onset hot flashes/sweating, grade ≥2		848 (43)	411 (42)	437 (45)
Early-onset musculoskeletal symptoms, grade ≥2		516 (26)	331 (34)	185 (19)
Side effect and time point	Grade			
Hot flashes				
Baseline	Unk	2 (0)	2 (0)	0 (0)
	Gr0	1812 (92)	924 (94)	888 (91)
	Gr1	134 (7)	51 (5)	83 (8)
	Gr2	19 (1)	10 (1)	9 (1)
3 months	Unk	8 (0)	3 (0)	5 (1)
	Gr0	637 (32)	325 (33)	312 (32)
	Gr1	766 (39)	386 (39)	380 (39)
	Gr2	500 (25)	248 (25)	252 (26)
	Gr3	56 (3)	25 (3)	31 (3)
6 months	Unk	20 (1)	9 (1)	11 (1)
	Gr0	525 (27)	294 (30)	231 (24)
	Gr1	797 (41)	401 (41)	396 (40)
	Gr2	573 (29)	260 (26)	313 (32)
	Gr3	52 (3)	23 (2)	29 (3)
Sweating				
Baseline	Unk	3 (0)	3 (0)	0 (0)
	Gr0	1832 (93)	920 (93)	912 (93)
	Gr1	119 (6)	56 (6)	63 (6)
	Gr2	13 (1)	8 (1)	5 (1)
3 months	Unk	9 (0)	4 (0)	5 (1)
	Gr0	1332 (68)	675 (68)	657 (67)
	Gr1	444 (23)	219 (22)	225 (23)
	Gr2	182 (9)	89 (9)	93 (9)
6 months	Unk	22 (1)	9 (1)	13 (1)
	Gr0	1285 (65)	688 (70)	597 (61)
	Gr1	447 (23)	206 (21)	241 (25)
	Gr2	213 (11)	84 (9)	129 (13)
Musculoskeletal symptoms				
Baseline	Unk	3 (0)	3 (0)	0 (0)
	Gr0	1669 (85)	837 (85)	832 (85)
	Gr1	248 (13)	125 (13)	123 (13)
	Gr2	47 (2)	22 (2)	25 (3)
	Gr3	0 (0)	0 (0)	0 (0)
3 months	Unk	9 (0)	5 (1)	4 (0)
	Gr0	1360 (69)	670 (68)	690 (70)
	Gr1	467 (24)	231 (23)	236 (24)
	Gr2	118 (6)	74 (7)	44 (4)
	Gr3	13 (1)	7 (1)	6 (1)
6 months	Unk	21 (1)	9 (1)	12 (1)
	Gr0	1070 (54)	463 (47)	607 (62)
	Gr1	648 (33)	366 (37)	282 (29)
	Gr2	198 (10)	128 (13)	70 (7)
	Gr3	30 (2)	21 (2)	9 (1)
9 months	Unk	41 (2)	24 (2)	17 (2)
	Gr0	995 (51)	386 (39)	609 (62)
	Gr1	670 (34)	392 (40)	278 (28)
	Gr2	228 (12)	159 (16)	69 (7)
	Gr3	33 (2)	26 (3)	7 (1)
12 months	Unk	37 (2)	23 (2)	14 (1)
	Gr0	965 (49)	388 (39)	577 (59)
	Gr1	708 (36)	400 (41)	308 (31)
	Gr2	235 (12)	158 (16)	77 (8)
	Gr3	22 (1)	18 (2)	4 (0)

Reports of hot flashes and sweating side effects and of musculoskeletal symptoms according to common terminology criteria for adverse events (CTCAE) v3.0 at time points during the first year of protocol therapy. All data are summarized as number (%) of patients. ^a^Patients without any adverse event data (two patients without hot flashes/sweating and one without musculoskeletal symptoms) were excluded from summary. *Unk* unknown, *Gr* grade *OFS* ovarian function suppression

### Association of *CYP19A1* and *ESR1* with early-onset hot flashes/sweating

A total of 43 % of patients reported early-onset grade 2–3 hot flashes/sweating during the first 6 months of protocol treatment (42 % of women receiving exemestane + OFS (411/987) and 45 % of women allocated to tamoxifen + OFS (437/980)). Most side effects were reported by the month-3 visit (Table 3). Overall, patients with *CYP19A1* rs10046 (C > T) minor variant (T/T) had a 22 % reduced odds of reporting early-onset grade 2–3 hot flashes/sweating (odds ratio (OR) = 0.78, 95 % CI 0.63–0.97; *P* = 0.03) when compared to patients with the C/T or C/C variants. The effect was consistent, showing a multivariable OR of 0.83 (95 % CI 0.66–1.04; *P* = 0.10) after adjusting for patient and treatment characteristics and concomitant medications (Table 4). A differential effect according to treatment assignment (treatment-by-genotype interaction, *P* = 0.03) was observed for the association between *CYP19A1* rs10046 (C > T) genotype variants and early-onset hot flashes/sweating. Patients treated with exemestane + OFS and having the T/T variant had a 35 % reduced odds of early-onset hot flashes/sweating (Table 5; univariate OR = 0.65, 95 % CI 0.48–0.89; multivariable OR = 0.67, 95 % CI 0.49–0.93), which was not apparent for patients treated with tamoxifen + OFS (univariable OR = 0.94, 95 % CI 0.69–1.27; multivariable OR = 1.04, 95 % CI 0.75–1.43). There was no statistically significant association between the other four SNPs of *CYP19A1* or *ESR1* and early-onset hot flashes/sweating side effects.

**Table 4:** Associations of *CYP19A1* and *ESR1* genotypes with analysis endpoints

			Univariate model		Multivariable^b^ model		Multivariable^c^ model	
Gene: SNP	Comparisons	Number^a^ (events)	Odds ratio (95 % CI)	*P* value	Odds ratio (95 % CI)	*P* value	Odds ratio (95 % CI)	*P* value
Hot flashes/sweating								
*CYP19A1*: rs4646	Dose effect^d^	1965 (848)	1.05 (0.91,1.21)	0.50	1.04 (0.90,1.20)	0.63	1.08 (0.93,1.25)	0.30
*CYP19A1*: rs10046	T/T vs. C/T,C/C (ref)	446 (172) vs. 1519 (676)	0.78 (0.63,0.97)	0.03	0.82 (0.66,1.02)	0.08	0.83 (0.66,1.04)	0.10
*ESR1*:rs2077647	Dose effect	1965 (848)	0.95 (0.84,1.08)	0.47	0.96 (0.84,1.09)	0.51	0.97 (0.85,1.11)	0.69
*ESR1*:rs2234693 (PvuII)	Dose effect	1965 (848)	0.92 (0.80,1.04)	0.18	0.92 (0.80,1.04)	0.19	0.94 (0.82,1.07)	0.36
*ESR1*:rs9340799 (XbaI)	Dose effect	1965 (848)	0.94 (0.82,1.08)	0.38	0.94 (0.82,1.07)	0.34	0.98 (0.85,1.12)	0.73
Musculoskeletal symptoms								
*CYP19A1*: rs4646	Dose effect^d^	1966 (516)	1.01 (0.86,1.18)	0.90	1.05 (0.89,1.24)	0.55	1.11 (0.93,1.31)	0.25
*CYP19A1*: rs10046	T/T vs. C/T,C/C (ref)	446 (110) vs. 1520 (406)	0.90 (0.70,1.15)	0.39	0.86 (0.66,1.10)	0.23	0.84 (0.65,1.09)	0.18
*ESR1*:rs2077647	Dose effect	1966 (516)	1.08 (0.94,1.25)	0.28	1.12 (0.96,1.30)	0.15	1.11 (0.95,1.29)	0.20
*ESR1*:rs2234693 (PvuII)	Dose effect	1966 (516)	1.03 (0.90,1.19)	0.65	1.07 (0.92,1.25)	0.37	1.06 (0.91,1.24)	0.47
*ESR1*:rs9340799 (XbaI)	Dose effect	1966 (516)	1.06 (0.91,1.23)	0.44	1.10 (0.94,1.29)	0.22	1.11 (0.94,1.30)	0.22

Analysis endpoints were early-onset (within 6 months) grade ≥2 hot flashes/sweating or (within 12 months) grade ≥2 musculoskeletal symptoms. ^a^Patients without any adverse event data, excluded from analyses (2 without hot flashes/sweating and one without musculoskeletal symptoms). ^b^Multivariable logistic regression model adjusted for characteristics: age, menstruation status, BMI, adjuvant chemotherapy use, treatment assignment, and presence of hot flashes/sweating at baseline or of musculoskeletal symptoms at baseline (according to endpoint). ^c^Multivariable model also adjusted for relevant concomitant medications prior to or continuing at baseline, and use during relevant time period for the endpoint. ^d^Dose effect: comparisons of variant (Var) allele groups: 0 (Var) vs. 1 (Var) vs. 2 (Var). *SNP* single nucleotide polymorphism

**Table 5:** Associations of endpoints with SNP *CYP19A1* rs10046 variants, overall and according to treatment assignments

		*CYP19A1:* rs10046			
Endpoint	Cohort	T/T vs T/C, C/C patients (events)	Univariate model OR (95 % CI)^a^	Univariate model Interaction *P* value^b^	Multivariable model OR (95 % CI)^c^
Hot flashes/sweating	All patients	446 (172) vs 1519 (676)	0.78 (0.63, 0.97)	0.03	0.83 (0.66, 1.03)
	Exemestane + OFS	227 (77) vs 759 (334)	0.65 (0.48, 0.89)		0.67 (0.49, 0.93)
	Tamoxifen + OFS	219 (95) vs 760 (342)	0.94 (0.69, 1.27)		1.04 (0.75, 1.43)
Musculoskeletal events	All patients	446 (110) vs 1520 (406)	0.90 (0.70, 1.15)	0.39	0.85 (0.66, 1.1)
	Exemestane + OFS	227 (75) vs 759 (256)	0.97 (0.71, 1.33)		0.96 (0.69, 1.32)
	Tamoxifen + OFS	219 (35) vs 761 (150)	0.77 (0.52, 1.16)		0.77 (0.51, 1.17)

^a^Estimates from univariate logistic regression model. ^b^ *P* value from test of rs10046 variants ((T/T) vs. T/C, C/C) by treatment interaction in logistic regression model (univariable) assessing association between the SNP variants and early-onset adverse events in the overall cohort. ^c^Adjusted for baseline characteristics: age, menstrual status, body mass index, adjuvant chemotherapy use, treatment assignment (for “all patients” cohort), baseline hot flashes/sweating or baseline musculoskeletal symptoms (according to endpoint) and prior to or baseline concomitant medications use and use during relevant time period for the endpoint (yes or no). *SNP* single nucleotide polymorphism, *OFS* ovarian function suppression, *OR* odds ratio, *CI* confidence interval

### Association of *CYP19A1* and *ESR1* with early-onset musculoskeletal symptoms

Within the first year of treatment, 26 % of patients reported early-onset grade 2–4 musculoskeletal symptoms (34 % of patients (331/987) assigned to exemestane + OFS and 19 % of patients (185/980) assigned to tamoxifen + OFS. There was no statistically significant association between any of the five SNPs of *CYP19A1* and *ESR1* and early-onset musculoskeletal side effects (Table 4), nor of treatment-by-genotype interaction. The presence of *CYP19A1* rs10046 (C > T) minor variant (T/T) was not associated with early-onset grade 2–4 musculoskeletal symptoms (univariate OR = 0.90, 95 % CI 0.70–1.15; *P* = 0.39; OR = 0.84, 95 % CI 0.65–1.09; *P* = 0.18 after adjusting for patient and treatment characteristics and concomitant medications (Table 4). There was no evidence of a differential effect according to treatment assignment (exemestane + OFS, OR = 0.97, 95 % CI 0.71–1.33 versus tamoxifen + OFS, OR = 0.77, 95 % CI 0.52–1.16, treatment-by-genotype interaction; *P* = 0.39 from the univariate model). The results were consistent after adjusting for patient and treatment characteristics and concomitant medications (exemestane + OFS, OR = 0.96, 95 % CI 0.69–1.32 versus tamoxifen + OFS, OR = 0.77, 95 % CI 0.51 − 1.17) (Table 5).

## Discussion

This study provides evidence that *CYP19A1* rs10046 variant carriers may face milder vasomotor symptoms under combined endocrine treatment. Notably, the effect was restricted to patients under OFS combined with exemestane (treatment-by-genotype interaction, *P* = 0.03) and not tamoxifen, after adjusting for patient characteristics and concomitant medications, including the selective serotonin-reuptake inhibitors known to reduce hot flashes/sweating.

This finding is in line with evidence from others, linking this SNP to enhanced aromatase activity and higher circulating estrogens [15, 17] and underscores a possible relationship between the effect of this variant polymorphism (T/T), hot flashes/sweating and exemestane activity. This result may in fact be related to less effective estrogen suppression by exemestane + OFS in these women as a consequence of higher circulating estrogens compared to patients with wild-type SNPs, although the exact mechanism by which this SNP may affect exemestane efficacy in suppressing the aromatase activity is not known. One study recently reported similar associations in postmenopausal patients with breast cancer [30] enrolled in the TEAM trial: *CYP19A1* variants linked with lower estrogen levels were associated with increased risk of early vasomotor and musculoskeletal symptoms under exemestane. The TEAM substudy, however, only included 27 % of the patients enrolled, less than two-thirds of patients, which represents a smaller proportion than is recommended by Simon et al. for evaluating predictive biomarkers [31].

The ELPh trial was designed to address genetic associations with toxicity-related discontinuation of AI therapy for breast cancer [32], including the SNP rs10046. The authors did not specifically report on vasomotor symptoms, but did not find any relationship between rs10046 and toxicity-related treatment discontinuation. In another study, in which the impact of *CYP19A1* SNPs with estrogen suppression during letrozole treatment was assessed, the degree of suppression was independent of the SNPs [33].

To our knowledge this is the first study to evaluate the associations between common germline polymorphisms of the *CYP19A1* and *ESR1* genes and early-onset side effects under combined endocrine treatment in premenopausal patients with hormone receptor-positive early breast cancer. The strength of this translational research is its considerable sample size of 1967 patients, which represents 74 % of women enrolled in TEXT. Furthermore, blood samples were collected specifically for this research, i.e., to investigate treatment tolerability and disease outcome. Women enrolled prior to the amendment were asked to re-consent, but 693 TEXT participants were not assessed due to the retrospective nature of blood collection. As a result, we may have missed some patients who discontinued treatment early, possibly due to treatment-related side effects.

The combined analysis of TEXT and SOFT [4] showed that adjuvant treatment with exemestane + OFS as compared with tamoxifen + OFS, significantly reduces the risk of recurrence. Although the overall incidence of adverse events and the quality of life were similar in the two treatment groups, between-group differences were observed with respect to specific symptoms. While vasomotor AEs (hot flashes and sweating) were quite frequent and evenly distributed amongst treatment groups, musculoskeletal AEs were more frequently reported in patients assigned to exemestane + OFS.

We did not observe any direct association between the *CYP19A1* SNPs and musculoskeletal symptoms, nor any interaction by endocrine treatment. This is in contrast with findings from the TEAM trial [30], but as mentioned they studied a very small proportion of patients. Furthermore, genotyping in that study was performed on DNA extracted from tumor samples. A cross-sectional study of patients receiving AIs [34] found that women carrying at least one 8-repeat allele of the tetranucleotide repeat polymorphism of *CYP19A1*, associated with higher estrogen concentrations, had lower odds of AI-associated arthralgia. Conversely, they also did not find any association between the rs10046 SNP and arthralgia.

Contrary to findings from case–control studies conducted in different treatment settings, i.e., postmenopausal or premenopausal women with breast cancer treated with tamoxifen alone, we found no association between the three *ESR1* polymorphisms and endocrine-mediated side effects (hot flashes/sweating and musculoskeletal symptoms). Postmenopausal Chinese patients with breast cancer carrying an *ESR1* rs2234693 CC genotype or rs9340799 AA genotype had an increased risk of AI-related musculoskeletal AEs [35]. In fact, several studies suggest that the effect of the *ESR1* polymorphisms on breast cancer risk is hormone-related and dependent on the woman’s hormonal context, showing statistically significant associations mainly in premenopausal women [23]. Likewise, an association with increased mammographic density [36] was shown only in women taking hormone replacement therapy. Possibly, the concurrent OFS by the GnRH analogue triptorelin masked the effect of these polymorphisms due to its complete estrogen deprivation effect. Thus, in the context of adjuvant combined endocrine treatment, these *ESR1* polymorphisms may be unlikely to exert their effect.

Musculoskeletal events are a common toxicity, leading to premature discontinuation of AI therapy [37]. In the TEXT-SOFT combined analysis, early cessation of protocol treatment was more frequent among patients receiving exemestane + OFS than among those receiving tamoxifen + OFS. Several studies have investigated the relationship between endocrine treatment efficacy and associated side effects in different settings. Recent findings support an inverse association between the reporting of early side effects under adjuvant endocrine treatment and breast cancer recurrence [38–40]. Vasomotor symptoms were associated with improved disease-free and overall survival in the TEAM trial [38] and reduced breast cancer recurrence in the ATAC trial [40], but not in the BIG 1–98 [41] and MA.27 trials [42]. Thus, we cannot exclude that the *CYP19A1* rs10046 (T/T) genotype might be associated with reduced exemestane + OFS efficacy: women with this polymorphism possibly lack complete estrogen suppression, despite receiving concomitant OFS. On the other hand, because the rs10046 polymorphism is located in a 3’ untranslated region, upstream of the coding sequence, it may interfere with aromatase transcription in a tissue-specific manner, depending on the transcriptional modulators present, thus influencing the degradation rate of the aromatase differently according to tissue and independently from circulating estrogen [9].

## Conclusions

This translational study within the TEXT trial for premenopausal patients with hormone-receptor-positive early breast cancer provides evidence that the *CYP19A1* rs10046 polymorphism may influence endocrine treatment side effects under combined endocrine therapy. The *CYP19A1* rs10046 variant favors lower incidence of hot flashes/sweating under exemestane plus ovarian function suppression treatment, suggesting endocrine-mediated effects that might enhance treatment adherence and potentially impact long-term treatment efficacy. No effect of any other tested SNPs was evident on hot flashes/sweating and no interaction on musculoskeletal symptoms emerged overall. Monitoring of musculoskeletal and bone events, known to occur later during treatment are warranted. Although our results must be considered hypothesis-generating, longer follow up will allow us to assess the clinical relevance of this finding, in particular its potential impact on disease outcome, and will be the subject of a future report after the TEXT results are further updated.

## Abbreviations

ADL: activities of daily living
AE: adverse event
AI: aromatase inhibitor
BMI: body mass index
CALGB: Cancer and Leukemia Group B
CTCAE: common terminology criteria for adverse events
CYP19A1: Cytochrome P450 19A1 gene
E + OFS: exemestane plus ovarian function suppression
EDTA: ethylenediaminetetetraacetic acid
ER: estrogen receptor
ESR1: estrogen receptor α gene
GnRHa: gonadotropin-releasing-hormone analogues
HWE: Hardy-Weinberg equilibrium
IBCSG: International Breast Cancer Study Group
IQR: interquartile range
ITT: intention to treat
NSAID: nonsteroidal anti-inflammatory drugs
OFS: ovarian function suppression
PCR: polymerase chain reaction
PgR: progesterone receptor
REMARK: Reporting recommendations for tumor marker prognostic studies
SNP: single nucleotide polymorphism
SOFT: Suppression of Ovarian Function Trial
T + OFS: tamoxifen plus ovarian function suppression
TEXT: Tamoxifen and Exemestane Trial
Unk: unknown
